# High-specific-power flexible transition metal dichalcogenide solar cells

**DOI:** 10.1038/s41467-021-27195-7

**Published:** 2021-12-09

**Authors:** Koosha Nassiri Nazif, Alwin Daus, Jiho Hong, Nayeun Lee, Sam Vaziri, Aravindh Kumar, Frederick Nitta, Michelle E. Chen, Siavash Kananian, Raisul Islam, Kwan-Ho Kim, Jin-Hong Park, Ada S. Y. Poon, Mark L. Brongersma, Eric Pop, Krishna C. Saraswat

**Affiliations:** 1 Department of Electrical Engineering, Stanford University, Stanford, CA 94305 USA; 2 Geballe Laboratory for Advanced Materials, Stanford University, Stanford, CA 94305 USA; 3 Department of Materials Science and Engineering, Stanford University, Stanford, CA 94305 USA; 4 Department of Electrical and Computer Engineering, Sungkyunkwan University, Suwon, 16419 Korea; 5 Department of Electrical and Systems Engineering, University of Pennsylvania, Philadelphia, PA 19104 USA; 6 SKKU Advanced Institute of Nanotechnology (SAINT), Sungkyunkwan University, Suwon, 16419 Korea; 7 Department of Applied Physics, Stanford University, Stanford, CA 94305 USA

**Keywords:** Devices for energy harvesting, Solar cells, Solar cells, Two-dimensional materials, Electronic properties and devices

## Abstract

Semiconducting transition metal dichalcogenides (TMDs) are promising for flexible high-specific-power photovoltaics due to their ultrahigh optical absorption coefficients, desirable band gaps and self-passivated surfaces. However, challenges such as Fermi-level pinning at the metal contact–TMD interface and the inapplicability of traditional doping schemes have prevented most TMD solar cells from exceeding 2% power conversion efficiency (PCE). In addition, fabrication on flexible substrates tends to contaminate or damage TMD interfaces, further reducing performance. Here, we address these fundamental issues by employing: (1) transparent graphene contacts to mitigate Fermi-level pinning, (2) MoO_*x*_ capping for doping, passivation and anti-reflection, and (3) a clean, non-damaging direct transfer method to realize devices on lightweight flexible polyimide substrates. These lead to record PCE of 5.1% and record specific power of 4.4 W g^−1^ for flexible TMD (WSe_2_) solar cells, the latter on par with prevailing thin-film solar technologies cadmium telluride, copper indium gallium selenide, amorphous silicon and III-Vs. We further project that TMD solar cells could achieve specific power up to 46 W g^−1^, creating unprecedented opportunities in a broad range of industries from aerospace to wearable and implantable electronics.

## Abstract (web summary)

Ultrathin transition metal dichalcogenides (TMDs) hold promise for next-generation lightweight photovoltaics. Here, the authors demonstrate the first flexible high power-per-weight TMD solar cells with notably improved power conversion efficiency.

## Introduction

Conventional silicon (Si) solar cells dominate the photovoltaics market with a market share of about 95% due to their low-cost manufacturing and reasonable power conversion efficiency (PCE)^1^. However, the low optical absorption coefficient and brittle nature of Si lead to degraded performance in ultrathin, flexible Si solar cells and therefore prevent their broader usage in applications demanding high power per weight (i.e., specific power, *P*_S_) and flexibility, for example in aerospace, transportation, architecture, and self-powered wearable and implantable electronics^2–10^.

Emerging semiconducting transition metal dichalcogenides (TMDs) exhibit excellent properties for such flexible high-specific-power photovoltaics. These include ultrahigh optical absorption coefficients up to one order of magnitude greater than conventional direct bandgap semiconductors, near-ideal band gaps for solar energy harvesting, and self-passivated surfaces^11–18^. In fact, ultrathin (<20 nm) TMDs can achieve near-unity, broadband, and omnidirectional absorption in the visible spectrum^15,16^. The wide range of TMD band gaps (~1.0–2.5 eV)^17^ are also well suited for highly efficient single-junction or double-junction tandem solar cells^13^. In addition, the dangling-bond-free surfaces of layered TMDs enable heterostructures without the constraint of lattice matching, offering abundant design choices for TMD photovoltaics^18^. According to realistic detailed balance models^13^, a PCE of ~27% can be achieved in ultrathin single-junction TMD solar cells, leading to extremely high *P*_S_ once implemented on lightweight flexible substrates^8^.

Despite these promising forecasts, there have not been any such demonstrations due to difficulties in reaching high PCE and integrating materials on flexible substrates. The PCE of TMD solar cells has typically not exceeded 2%^19–25^, mostly due to strong Fermi-level pinning at the metal contact–TMD interface^26^ and the inapplicability of traditional doping schemes such as diffusion or ion implantation, which can damage TMDs^27^. Reducing or eliminating Fermi-level pinning by adopting a gentle metal transfer method^22,26^, introducing an ultrathin interlayer at the metal–TMD interface^28–31^, or forming a van der Waals (vdW) heterojunction such as graphene-TMD^32–34^ can significantly improve the performance of TMD devices. In addition, forming a p–n homojunction by employing TMD-compatible doping methods such as surface charge transfer and fixed charge doping via metal oxides^23–25^, plasma doping^35^, or electrostatic doping^36^ has been shown to enhance the photovoltaic performance. Noteworthily, the highest PCEs in thin-film single-junction TMDs are 2.8% in plasma-doped MoS_2_ and 6.3% in electrostatically-doped MoSe_2_ solar cells^35,36^. At the same time, TMDs are typically transferred to flexible substrates and most of these processes can damage TMD interfaces, leave unwanted polymer residues, and do not allow for a reliable and practical vertical device architecture^37^. Previous reports on *P*_S_ of TMD solar cells, i.e., 3 W g^−1^ with a PCE of 0.46%^22^ and 2500 W g^−1^ with a PCE of 1.0%^14^, do not account for the substrate’s weight, which practically constitutes the largest part of the overall weight and needs to be considered for accurate *P*_S_ calculations. The only TMD solar cell on a lightweight, flexible substrate reported to date has a PCE of <0.7%, yielding a *P*_S_ of <0.04 W g^−1^ ^20^.

Here, we address the above-mentioned device and integration challenges by utilizing transparent graphene contacts mitigating Fermi-level pinning, MoO_*x*_ capping for doping, passivation and anti-reflection coating, and a clean, non-damaging direct transfer method to realize TMD solar cells for the first time on an ultrathin (5 μm), lightweight and flexible polyimide (PI) substrate. The flexible TMD (WSe_2_) solar cells made in this fashion achieve a PCE of 5.1%, surpassing previous flexible TMD solar cells by more than an order of magnitude^20^. Furthermore, the integration on an ultrathin substrate enables a *P*_S_ of 4.4 W g^−1^, more than 100× higher than previous results on flexible TMD photovoltaics^20^ and in the same range as champion solar cells of prevailing thin-film technologies cadmium telluride (CdTe), copper indium gallium selenide (CIGS), amorphous silicon (a-Si) and group III–V semiconductors^38–45^. In future, TMD solar cells on even thinner substrates and with higher PCEs could potentially achieve an unprecedented *P*_S_ of ~46 W g^−1^ (as we project in this work) opening up far-reaching possibilities in a broad range of industries^9^.

## Results and discussion

### Design and fabrication of flexible WSe_2_ solar cells

**Fig. 1:**
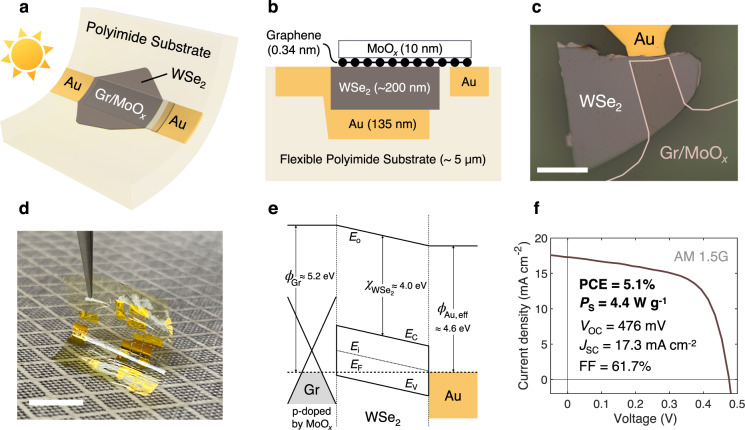
Flexible WSe_2_ solar cells. **a** Device schematic and **b** cross-section. **c** Top-down optical image of the device. Scale bar, 50 μm. **d** Photograph of WSe_2_ solar cells on a flexible polyimide substrate. Scale bar, 1 cm. **e** Qualitative energy band diagram of the device. The Fermi level (*E*_F_) is pinned near the midgap at the Au–WSe_2_ interface, but not at the van der Waals graphene (Gr)–WSe_2_ interface^26,32,33,50,51^. MoO_*x*_ increases the Gr work function and the built-in potential of the Gr–WSe_2_ Schottky junction^53^. *E*_0_, vacuum level; *E*_C_, conduction band edge; *E*_V_, valence band edge; *E*_i_, intrinsic Fermi level; *Φ*_Au, eff_, Au effective work function; *Φ*_Gr_, Gr work function; ${\chi }_{{{{{{{\rm{WSe}}}}}}}_{2}}$, electron affinity of WSe_2_. **f** Measured current density *J* vs. voltage *V* under AM 1.5 G illumination. PCE, power conversion efficiency. *P*_S_ specific power or power per weight. *V*_OC_ open-circuit voltage. *J*_SC_ short-circuit current. FF fill factor.

We fabricate flexible vertical photovoltaic cells from multilayer (~200 nm) tungsten diselenide (WSe_2_) absorbers, transparent hole-collecting graphene top contacts covered by MoO_*x*_ doping, passivation and anti-reflection coatings, and optically-reflective electron-collecting gold (Au) bottom contacts. The bottom contact and absorber material are embedded into a flexible, transparent polyimide substrate. Device schematics and optical images are shown in Fig. 1a–d. We mechanically exfoliate WSe_2_ flakes on thermally oxidized silicon substrates and deposit patterned Au bottom contacts, which are all covered with spin-coated polyimide and released together in deionized water^46^. The patterned transparent top contacts constituted of graphene and MoO_*x*_ are then formed on top via graphene wet transfer and MoO_*x*_ electron-beam (e-beam) evaporation. Details on device fabrication and transfer procedures can be found in Supplementary Note 1 and Supplementary Fig. 1.

Figure 1e shows the schematic energy band diagram of flexible WSe_2_ solar cells based on energy levels of WSe_2_, graphene (Gr), and Au reported in the literature. WSe_2_ has a bulk band gap of ~1.2 eV and electron affinity of ~4.0 eV^21,47–49^, and is undoped according to the bulk crystal vendor. Due to the energetic nature of e-beam evaporation, defect states are induced at the Au–WSe_2_ interface, and the Au Fermi level is pinned toward the charge neutrality level of WSe_2_ located around midgap^26,50,51^. This decreases the effective work function of Au and makes it a decent electron-collecting contact. We find that replacing Au with lower work function metals such as Ti and Al leads to a lower performance, most probably due to their reactive nature therefore forming poor interfaces with WSe_2_ (Supplementary Fig. 2)^52^. On the other hand, layered materials Gr and WSe_2_ experience no Fermi-level pinning at their vdW interface^32–34^. The work function of undoped graphene is ~4.6 eV (e.g., in vacuum), which increases to ~5.0 eV when graphene is exposed to air^53–55^. Graphene and the undoped WSe_2_, therefore, form a Schottky junction with a hole barrier height of 0.1–0.2 eV and a built-in potential of 0.4–0.5 eV^33,34,56^. The MoO_*x*_ on top further p-dopes graphene, increasing its work function and therefore the built-in potential of the Gr–WSe_2_ Schottky junction by ~0.16 eV (Supplementary Note 2, Supplementary Table 1 and Supplementary Fig. 3a, b). MoO_*x*_ also passivates the top surface of the solar cell, specifically the trap states at the Gr–WSe_2_ interface^24^. These lead to a higher open-circuit voltage (*V*_OC_) and short-circuit current density (*J*_SC_) in MoO_*x*_-capped WSe_2_ solar cells (Supplementary Note 2 and Supplementary Fig. 3c). As we will discuss later in the optical characterization section, MoO_*x*_ also serves as an effective anti-reflection coating for WSe_2_, leading to an additional increase in *J*_SC_. Given the approximate locations of Gr, WSe_2_, and Au Fermi levels, the depletion regions of Gr–WSe_2_ and Au–WSe_2_ Schottky junctions are estimated to be in the order of 1 μm and therefore expand throughout the entire depth of the ~200-nm-thick WSe_2_ layer, leading to fully depleted devices with a built-in potential of ~0.6 eV.

### Photovoltaic performance

Under global air mass AM 1.5 G illumination, the flexible WSe_2_ solar cells achieve *V*_OC_ of 476 mV, a *J*_SC_ of 17.3 mA cm^−2^, and a fill factor (FF) of 61.7% (Fig. 1f), leading to an unprecedented PCE of 5.1% in flexible TMD solar cells, over 10× higher than previous demonstrations (<0.7%)^20^. Having an ultrathin absorber layer and a lightweight polyimide substrate, these WSe_2_ solar cells also achieve a high-specific power (*P*_S_) of 4.4 W g^−1^ (calculated in Supplementary Note 3 using the data in Supplementary Table 2), over 100× higher than preceding results (<0.04 W g^−1^)^20^ and on par with champion solar cells from well-established thin-film technologies CdTe, CIGS, a-Si, and III-Vs^38–45^. The device has a shunt resistance of 226 Ω cm^2^ and a series resistance of 3.1 Ω cm^2^, calculated by inversing the slope of the *J*−*V* curve at short-circuit and open-circuit conditions, respectively, yielding the reasonable FF of 61.7%. We measure reproducible performance in all nine devices fabricated (Supplementary Fig. 4). No hysteresis is observed in the *J*–*V* characteristics when sweeping the voltage in the forward and backward directions (Supplementary Fig. 5). Similar *J*–*V* characteristics are observed in solar cells with undoped tungsten disulfide (WS_2_) absorber layers. However, due to their lower built-in potential, WS_2_ solar cells exhibit lower *V*_OC_, *J*_SC_, FF, and hence PCE (Supplementary Fig. 6).

### Electrical characterization

**Fig. 2:**
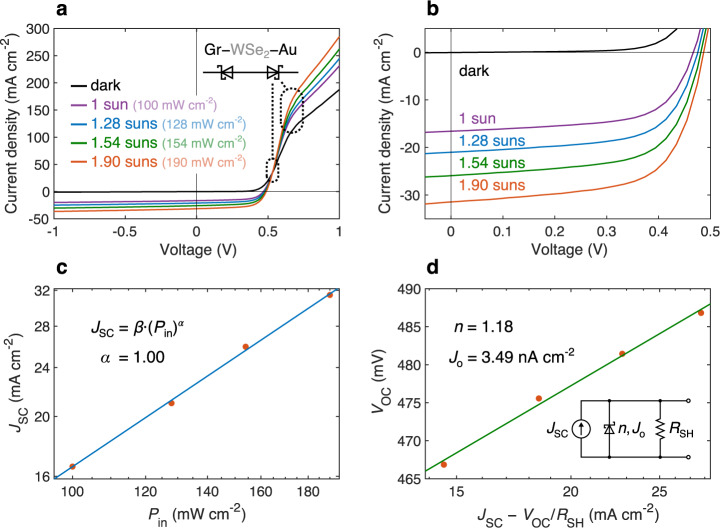
Electrical characteristics of flexible WSe_2_ solar cells. **a** Current density vs. voltage (*J–V*) characteristics of WSe_2_ solar cells under AM 1.5 G illumination, at various incident power. Inset represents the circuit diagram of Au–WSe_2_ and Gr–WSe_2_ junctions. With its Fermi level pinned around midgap, Au forms a Schottky junction to WSe_2_ opposing the main Gr–WSe_2_ Schottky junction, leading to cross-over and roll-over effects occurring around 0.53 and 0.65 V, respectively^57,58^, as marked by dotted ovals. **b** Zoomed-in view of the photovoltaic region of the *J–V* measurements shown in **a**. **c** Short-circuit current density (*J*_SC_) of the devices. *P*_in_, incident power; symbols, measurements; line, power law fit. **d** Open-circuit voltage (*V*_OC_) of the devices. Note logarithmic axes in **c** and horizontal axis in **d**. *R*_SH_, shunt resistance; symbols, measurements; line, fit. Inset shows a representative circuit diagram. *n* is the ideality factor and *J*_o_ the dark saturation current density from the diode fit.

Next, we measure the current density vs. voltage (*J–V*) characteristics of flexible WSe_2_ solar cells in the dark and for AM 1.5 G illumination at various incident power intensities (Fig. 2a, b). As shown in Fig. 1e, Au is not an ohmic n-type contact and, similar to Gr, forms a Schottky junction with WSe_2_, resulting in a back-to-back diode structure (Fig. 2a, inset). This undesirable Schottky barrier at the Au back contact leads to the roll-over phenomenon where the slope of the *J*–*V* curve is reduced at high forward biases^57,58^, starting here at around *V* = 0.65 V. The barrier also causes the cross-over of dark and light *J*–*V* curves at *V* = 0.53 V, which can be explained by the presence of a minority carrier surface recombination current at the Au–WSe_2_ interface^58^. The two phenomena are also frequently observed in CdTe and CIGS solar cells^57–59^.

Figure 2b shows a zoomed-in view of the photovoltaic region. An analysis of this data indicates that the shunt resistance decreases almost linearly with increasing incident intensity (Supplementary Fig. 7). This phenomenon, known as photoshunting, occurs due to increased minority carrier conductivity across the device under illumination^60,61^. Improving the charge carrier selectivity of the solar cell, for example by utilizing carrier-selective metal-interlayer-semiconductor (MIS) contacts or introducing a high built-in potential p–n homojunction could reduce or eliminate photoshunting. Given the initially high shunt resistance of the device, photoshunting does not affect the shape of the *J–V* curve, and therefore fill factor stays constant at various intensities.

By fitting a power-law equation on the measured current density and incident power data (Fig. 2c), we observe that short-circuit current density versus incident power follow a linear trend (*J*_SC_ = *β*·(*P*_in_)^*α*^, *α* = 1), expected from a well-designed solar cell. Equation 1 is a rearrangement of the diode equation in the presence of photogeneration (*J*_photo_ = *J*_SC_) and shunt resistance (*R*_SH_) at *V* = *V*_OC_, leading to zero current density by definition (*J* = 0). In this equation, *n* is the diode ideality factor, *k*_B_ is the Boltzmann constant, *T* is the absolute temperature, *q* is the elementary charge, and *J*_o_ is the dark saturation current. According to this equation, *V*_OC_ scales linearly with ln(*J*_SC_ – *V*_OC_/*R*_SH_) when (*J*_SC_ – *V*_OC_/*R*_SH_)/*J*_o_ ≫ 1, valid for the WSe_2_ solar cells in this study. By fitting the measured *V*_OC_ and *J*_SC_ – *V*_OC_/*R*_SH_ (Fig. 2d), we extract *n* and *J*_o_ of the WSe_2_ solar cells, neglecting the Au back Schottky diode for simplicity.

WSe_2_ solar cells demonstrate a desirable near-unity ideality factor of *n* = 1.18 and dark saturation current of *J*_o_ = 3.49 nA cm^−2^. The near-unity ideality factor and small dark saturation current indicate low levels of charge carrier recombination and therefore a high internal quantum efficiency as confirmed later by comparing the measured *J*_SC_ and *J*_SC, max_ derived from absorption measurements.

1 $${V}_{{{{{{{\mathrm{OC}}}}}}}}=\frac{n{k}_{{{{{{\mathrm{B}}}}}}}T}{q}{{{{{\rm{ln}}}}}}\left(\frac{{J}_{{{{{{{\mathrm{SC}}}}}}}}-\frac{{V}_{{{{{{{\mathrm{OC}}}}}}}}}{{R}_{{{{{{{\mathrm{SH}}}}}}}}}}{{J}_{{{{{{\mathrm{o}}}}}}}}+1\right)$$

### Optical characterization and simulation

**Fig. 3:**
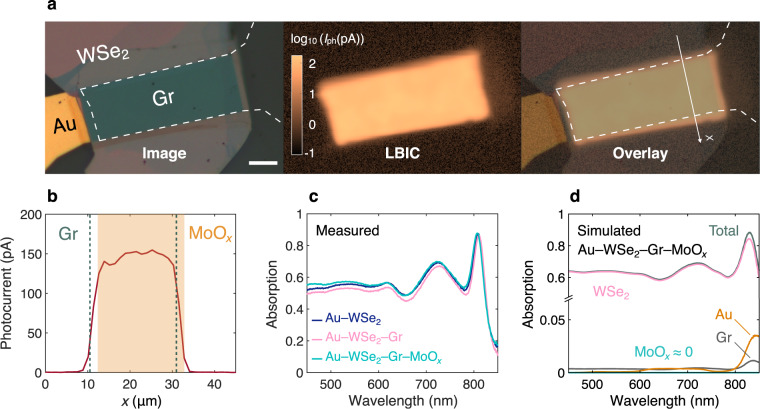
Optical characteristics of flexible WSe_2_ solar cells. **a** Optical image (left), light beam-induced current (LBIC or photocurrent) map (middle), and their overlay (right) for a typical WSe_2_ solar cell, measured at *λ* = 530 nm. Scale bar, 10 μm. **b** Photocurrent profile along the *x* axis shown in **a**, demonstrating that the Gr/MoO_*x*_–WSe_2_ junction area accurately represents the active area of the solar cell. The misaligned MoO_*x*_ is passivating the WSe_2_ top surface^24^, leading to the current generation up to the edge of MoO_*x*._ The photocurrent tails beyond the edges of Gr and MoO_*x*_ occur due to the finite laser spot size (~2 μm). In most devices, MoO_*x*_ and Gr are fully aligned like in Fig. 1c. **c** Measured absorption spectra at the center of the active area, taken at various stages of the fabrication process: after polyimide release (only with Au–WSe_2_), Gr transfer, and MoO_*x*_ deposition. **d** Simulated absorption spectra of the same device, matching well with the measurement in **c**. The plot also shows the contribution of each layer to the overall absorption. Nearly all absorption occurs within the 209-nm-thick WSe_2_ absorber layer. Note the discontinuous vertical axis, used to magnify the smaller contributions.

Figure 3a shows the optical image, spatial light beam-induced current (LBIC or photocurrent) map acquired at a wavelength of 530 nm, and their overlay for a typical flexible WSe_2_ solar cell. The overlay map shows that only the Gr–WSe_2_ diode is responsible for splitting photogenerated holes and electrons and therefore producing photocurrent. No photocurrent generation is observed at the Au–WSe_2_ back diode. This can be seen at the narrow WSe_2_ region near the Au contact line on the left, where the Au bottom contact is present, but no Gr is covering the WSe_2_. In contrast, a strong photocurrent is measured on the opposite side of the WSe_2_ on the right, where the Au back contact is absent and WSe_2_ is only in contact with Gr. This is further visualized by a cross-shaped contact scheme in Supplementary Fig. 8.

To accurately define the active area of the device, the photocurrent profile across the width of the device (*x* axis in Fig. 3a) is plotted on a linear scale (Fig. 3b). In this specific device, MoO_*x*_ is slightly misaligned with respect to Gr (Fig. 3a). The misalignment only occurred in few devices due to lithography issues. Most devices, such as the one shown in Fig. 1f, have well-aligned Gr and MoO_*x*_. On the left edge of Fig. 3b (corresponds to the upper edge in the photocurrent map), photocurrent is only generated in regions covered by Gr. The tail beyond the Gr edge occurs due to the finite laser spot size (~2 μm), leading to spatial averaging of the photocurrent. This spatial averaging shows up as a ~2-μm tail going from non-zero to zero photocurrent regimes as visible in the photocurrent profile. On the right side of Fig. 3b (corresponds to the lower edge in the photocurrent map), due to the passivation effect of MoO_*x*_^24^, current generation goes beyond Gr and occurs up to the MoO_*x*_ right edge. A similar spatial averaging phenomenon is also taking place on this side, resulting in a ~2-μm tail beyond the MoO_*x*_ edge. The photocurrent profile confirms that photogeneration only occurs in regions covered by Gr (and MoO_*x*_, if misaligned) and this area can be used to accurately define the active area of the device for current density calculation, similar to other studies on vertical TMD solar cells^22^. The active area of the solar cells tested varies from ~10^3^ to ~10^4^ μm^2^.

We measure the absorption spectrum of WSe_2_ solar cells at different stages of fabrication, i.e., after polyimide release (Au–WSe_2_), after Gr transfer (Au–WSe_2_–Gr), and finally after MoO_*x*_ deposition (Au–WSe_2_–Gr–MoO_*x*_), as shown in Fig. 3c. For consistency, each measurement is taken at exactly the same spot at the center of the active area of the device. The data in Fig. 3c corresponds to the device whose *J–V* characteristics are shown in Fig. 1f. This device has a 209-nm-thick WSe_2_ absorber layer, as measured by a stylus-based surface profiler.

After transferring Gr on top of WSe_2_, the overall absorption of the stack is slightly reduced. Optical simulations using the transfer matrix method produce a similar result (Supplementary Fig. 9a). Depositing 10 nm of MoO_*x*_ on top of Gr increases the overall absorption of the stack. This can be either due to parasitic absorption within MoO_*x*_ or its anti-reflection coating effect improving the absorption within the WSe_2_ absorber layer. To answer this question, we simulate absorption using the transfer matrix method. Figure 3d shows a simulated absorption spectrum of the Au–WSe_2_–Gr–MoO_*x*_ stack along with the contribution of each individual layer. Simulated and measured absorption spectra are in good agreement, having the same shapes and magnitudes, with peaks and valleys located at similar wavelengths. The small discrepancies between the two absorption spectra can be explained by the fact that the optical properties of WSe_2_ used in the simulation are taken from the literature^62^ and may deviate slightly from the WSe_2_ films in this study.

According to Fig. 3d, parasitic absorption in the 10-nm MoO_*x*_ layer is essentially zero. In addition, nearly all absorption occurs within the 209-nm-thick WSe_2_ absorber layer, indicating that the absorption boost observed after MoO_*x*_ coating (Fig. 3c) is mainly due to the increased absorption in the WSe_2_ layer. These observations suggest that MoO_*x*_ is acting as an anti-reflection coating for the WSe_2_ absorber layer. Our optical simulation confirms this hypothesis (Supplementary Fig. 9b, c), showing that adding MoO_*x*_ increases the absorption within the WSe_2_ layer. The simulation also reveals that an optimal choice of MoO_*x*_ thickness (~70 nm) can lead to a significant improvement in WSe_2_ absorption, resulting in *J*_SC_ values up to 30 mA cm^−2^ (Supplementary Fig. 9c, d). This suggests that MoO_*x*_ could be used as a simple yet effective anti-reflection coating choice for TMD photovoltaics, to be further investigated in future studies.

The WSe_2_ solar cells show an average optical absorption of about 55% over the 450–850 nm wavelength spectrum. Using the simulated WSe_2_ absorption spectrum and assuming unity internal quantum efficiency (IQE), we calculate a maximum *J*_SC_ of 20.0 mA cm^−2^, slightly underestimated because absorption at wavelengths below 400 nm and above 1000 nm are excluded due to lack of available material data (see Supplementary Fig. 9d). Given the measured *J*_SC_ of 17.3 mA cm^−2^ (Fig. 1f), this implies an average IQE (weighted by AM 1.5 G spectrum) of 0.87, which signals low levels of charge carrier recombination, in agreement with near-unity ideality factor and small dark saturation current extracted from *J–V* measurements (Fig. 2d). Similar IQE values have been observed in other vertical Schottky junction TMD solar cells^22^.

### Bending test

**Fig. 4:**
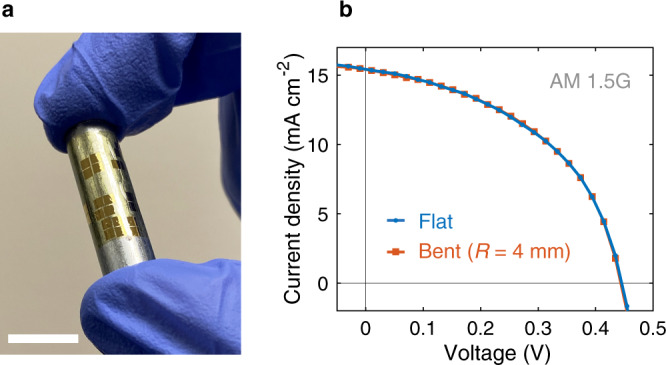
Bending test. **a** Photograph of the bending setup. The polyimide substrate is attached to an 8-mm-diameter metal cylinder, causing bending of the substrate at a curvature radius of 4 mm. Scale bar, 1 cm. **b** Measured *J–V* characteristics of a typical flexible WSe_2_ solar cell under AM 1.5 G illumination in flat and bent conditions. Bending does not change the *J–V* characteristics of the device.

To test the performance of devices under bending, we attach the polyimide substrate onto an 8-mm-diameter metal cylinder, which bends the substrate at a curvature radius of 4 mm (Fig. 4a). The flexible WSe_2_ solar cells show the same *J–V* characteristics in flat and bent states under AM 1.5 G illumination (Fig. 4b), indicating consistent performance levels under bending. This is not surprising because given the polyimide substrate thickness of only 5 μm, the materials encounter small strain values of ~0.06% at this bending radius^46^, and we expect that sub-millimeter bending radii should be possible given that materials involved have been shown to sustain strains of at least 0.5%^63–68^. We have investigated similar TMD, metal, and dielectric stacks in electronic devices in more detail in our recent work and found that there is no discernable change of electrical device properties^46^. Scaling up the solar cell area will not change the device stability under strain^63,69^, however, we expect effects of substrate curvature on light-coupling to become relevant. For instance, it has been found that *J*_SC_ and PCE reduce^70–72^ by ~30% under an incident angle of 45°. With the bending radius of 4 mm, this would mean that a ~6.3 mm wide solar cell would encounter such a 30% reduction at its outermost edges. Hence, for our solar cells with small, exfoliated flakes on length scales that are tens of microns, there is no effect of substrate curvature on the light-coupling, as seen in Fig. 4b. In the future, if the active area of the solar cell is increased, e.g., by large-area synthesis of TMDs, these bending studies will become more important to quantify the effects of the bent surface on light-coupling and thus *J*_SC_, which would alter solar cell performance.

### Benchmarking and projections

**Fig. 5:**
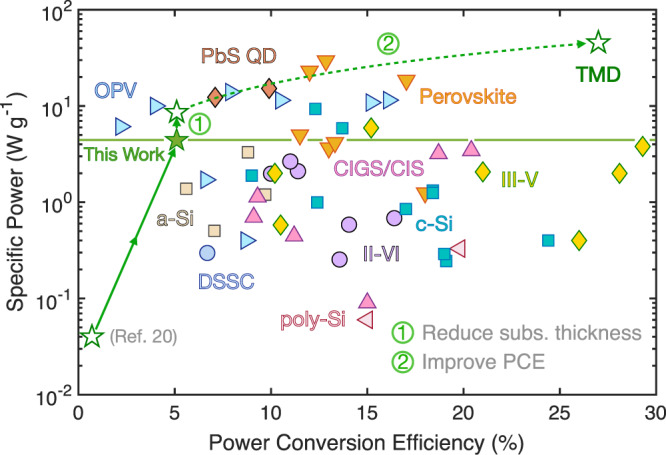
Power conversion efficiency (PCE) and specific power (power per weight) of lightweight and flexible thin-film solar technologies. Our flexible TMD (WSe_2_) solar cell achieves a relatively high specific power despite its moderate PCE (filled green star). Arrow 1 shows the projected effect of reducing substrate thickness, arrow 2 shows the projected effect of improving PCE. With these improvements, TMD solar cells could reach unprecedented specific power in the future. More details are given in Supplementary Note 4. OPV organic photovoltaics, PbS QD lead sulfide quantum dot, CIGS/CIS copper indium (gallium) selenide, c-Si crystalline silicon, a-Si amorphous silicon, poly-Si polycrystalline silicon, DSSC dye-sensitized solar cell, III–V (II–VI) compound semiconductors containing elements from groups three and five (or two and six) in the periodic table (see Supplementary Table 3 for more details and references).

Figure 5 benchmarks the PCE and *P*_S_ of the flexible WSe_2_ solar cells in this work against other thin-film solar technologies (details in Supplementary Note 4). The flexible WSe_2_ solar cells in this study demonstrate remarkable improvements of about 10× and 100× in PCE and *P*_S_, respectively, compared to the previous results in flexible TMD solar cells (PCE < 0.7% and *P*_S_ < 0.04 W g^−1^)^20^. With only a moderate PCE of 5.1%, the WSe_2_ solar cells already achieve a high-specific power of 4.4 W g^−1^ enabled by their ultrathin WSe_2_ absorber layer and lightweight polyimide substrate. This high *P*_S_ is in the same range as champion solar cells of well-established thin-film technologies CdTe (II-VI), CIGS, a-Si, and III-Vs^38–45^.

By reducing the polyimide substrate thickness to 1 μm, same as in some of the champion organic PV (OPV) and perovskite solar cells in Fig. 5, specific power can be further increased to 8.6 W g^−1^ (path #1 in Fig. 5). According to realistic detailed balance models developed for TMD photovoltaic cells, single-junction multilayer TMDs can in principle achieve ~27% PCE with an optimized optical and electronic design^13^. We have estimated similar values specifically for WSe_2_ solar cells as shown in Supplementary Note 5 and Supplementary Fig. 10. Such PCE would lead to an ultrahigh specific power of 46 W g^−1^ (path #2 in Fig. 5), by far outperforming all other thin-film technologies, including perovskite solar cells which currently hold the record for specific power (29.4 W g^−1^)^73^. In addition, TMD solar cells do not have the stability challenges of OPV or perovskites, and in contrast to high-performing perovskites and lead sulfide (PbS) quantum dots, they do not contain toxic elements such as lead, and therefore are not expected to pose any significant environmental or health hazards^74^.

### Future research

In order to achieve the projected ~27% PCE in the flexible WSe_2_ solar cells, both optical and electronic designs need to be improved. As pointed out earlier, MoO_*x*_ can serve as an effective anti-reflection coating for WSe_2_. Our optical simulation shows that simply increasing the thickness of MoO_*x*_ to an optimal value of ~70 nm is supposed to improve the absorption within the WSe_2_ layer to 80% and enable *J*_SC_ values up to 30 mA cm^−2^ (Supplementary Fig. 9c, d). Metasurface-based plasmonic light-trapping schemes can help further improve absorption and reach *J*_SC_ values near the Shockley–Queisser limit (40 mA cm^−2^ for a bandgap of 1.2 eV)^75–79^.

*V*_OC_ is another important area of improvement. The built-in potential and therefore *V*_OC_ of these devices can be improved by employing n-type WSe_2_. The doping process can be performed during growth, or by means of ultrathin metal-oxide interlayers such as AlO_*x*_ and TiO_*x*_ placed between the TMD absorber layer and the metallic bottom contact^80,81^. Replacing Au with a lower work function metal but ensuring a similar interface quality could also improve *V*_OC_. We showed in our experiments that Al and Ti are not good candidates for this purpose (Supplementary Fig. 2). Forming a high built-in potential p–n heterojunction (e.g., WSe_2_/MoS_2_) or homojunction, possibly by metal-oxide-based p-type (MoO_*x*_) and n-type (AlO_*x*_) doping, is another way to achieve a high *V*_OC_^23–25,80,82,83^. One can also improve the *V*_OC_ by adopting carrier-selective (MIS) contacts which both de-pin the Fermi level and enable a selective collection of only one type of charge carrier on each side of the solar cell^28–31,84,85^.

Research efforts to scale up TMD growth to large areas would soon enable scalable and low-cost production of TMD photovoltaic cells^86,87^, similar to other chalcogenide solar cells CdTe and CIGS. The potential of TMDs to achieve high power conversion efficiency and specific power at a low cost as well as their stability and environmentally friendliness (in contrast to perovskites) makes them a serious candidate for next-generation photovoltaics, especially in high-specific-power applications.

In summary, we demonstrated flexible WSe_2_ solar cells with record-breaking power conversion efficiency PCE of 5.1% and power per weight *P*_S_ of 4.4 W g^−1^. We performed detailed optical and electrical characterizations on these solar cells to explain their superior performance and identify areas of improvement, providing practical guidelines on the optical and electronic design to enhance PCE and *P*_S_. We also tested the flexible solar cells under bending and showed similar performance in flat and bent states. Future large-area TMD cells will require more bending studies to verify the effects of light-coupling at different angles on solar cell performance. Lastly, we benchmarked the flexible WSe_2_ solar cells against other thin-film photovoltaic technologies and showed their potential to achieve ultrahigh *P*_S_, creating unprecedented opportunities in a broad range of industries from aerospace to wearable electronics.

## Methods

### Device fabrication

The details on device fabrication are provided in Supplementary Note 1. In short, WSe_2_ flakes were mechanically exfoliated onto Si/SiO_2_ substrates. Next, 135 nm of Au serving as solar cell bottom contacts was electron-beam evaporated and structured by optical photolithography and lift-off. Then the flexible polyimide substrate was spin-coated on top, cured and all structures were released together in deionized water. After flipping the substrate, graphene was wet-transferred as transparent top contact and structured by optical photolithography and dry etching. Finally, MoO_*x*_ was deposited by electron-beam evaporation and structured by optical photolithography and lift-off, followed by annealing in ambient air for the purpose of doping, passivation, and anti-reflection coating. The process flow is schematically shown in Supplementary Fig. 1. The thickness of WSe_2_ flakes was measured by atomic force microscopy (AFM, Bruker Dimension Icon) and/or stylus-based surface profilometry (Alphastep 500) after exfoliation on the Si/SiO_2_ substrate.

### AM 1.5 G current–voltage measurements

AM 1.5 G *I*–*V* measurements were done using a digital source meter (Keithley 2420) and a class AAA solar simulator (Newport, Oriel Sol3A Class AAA) having a 450 W xenon short arc lamp and AM 1.5 G spectral correction filter. Lamp intensity calibration was done using a silicon reference cell (Newport, Oriel 91150 V) placed at the location of the sample. The silicon reference cell was calibrated by Newport Corporation. *I−V* characteristics were measured with a scan rate of 200 mV s^−1^ and a dwell time of 30 ms. The measurements were performed in air. The samples were kept at room temperature via convection cooling provided by a fan. In all *I*–*V* measurements, the voltage was applied to the graphene top contact, while the gold bottom contact was grounded.

### Photocurrent mapping

The photocurrent from the device was measured on a custom-built optoelectronic setup. A supercontinuum laser source (Fianium) and an acousto-optic tunable filter (Fianium) were used to provide monochromatic illumination across a broad spectral range. To achieve a high signal-to-noise ratio, the laser light was modulated by a chopper wheel (400 Hz) synchronized with two lock-in amplifiers. The laser light was focused onto the sample using a 50X long working distance objective (Mitutoyo M Plan APO NIR). To image the sample, two beam splitters were placed in the illumination path for a halogen lamp and a charge-coupled device (CCD) imaging camera, respectively. Using a glass slide, a small fraction of the reflected light was directed into a large-area Si photodiode (New Focus, model 2031) connected to a lock-in amplifier (Stanford Research SR810 DSP) to measure reflection from the sample. The sample was placed on a chip carrier and then mounted on a three-axis piezo stage to accurately control its spatial position. Electrical connections were made by soldering wires (34-gauge enameled wires) using thermally stable solder paste Sn42/Bi57.6/Ag0.4 T4 (Chip Quik) onto the Au metal pads. To measure the photocurrent, the mounted sample was connected in series with a source meter (Keithley 2612), a tunable current-to-voltage amplifier, and a second lock-in amplifier (Stanford Research SR810 DSP).

### Absorption measurement

The absorption measurements were performed using a Nikon C2 confocal microscope coupled to a CCD camera (Acton Pixis 1024, Princeton Instrument) and a spectrometer (Acton SP2300i, Princeton Instrument). Unpolarized light from a halogen lamp was used to illuminate the sample through a 20X objective (Nikon CFI Achromat LWD, NA = 0.4). The reflection spectra (*R*(λ)) were normalized to the calibrated reflection spectrum of a protected silver mirror (Thorlabs, PF10-03-P01). Since all devices were fabricated on top of thick Au contact, the absorption spectra (*A*(λ)) were calculated by *A*(λ) = 1 – *R*(λ).

### Raman spectroscopy

The Raman measurements were performed on a HORIBA Scientific LabRAM HR Evolution spectrometer using an excitation wavelength of 532 nm. For Raman measurements, an acquisition time, accumulations, laser power, and the optical grating of 20 s, 2, 2.8 mW, 1800 gr mm^−1^ were used, and the spot size is <1 μm.

### Optical simulation

Optical simulations were performed using the transfer matrix method. A normally incident plane wave was assumed in all simulations. The thickness of each layer used in simulations is shown in Fig. 1b. The bottom Au contact was modeled as a semi-infinite substrate due to its small penetration depth. Optical constants for WSe_2_, Gr, and Au were taken from the literature^62,88,89^. Optical constants for MoO_*x*_ were obtained experimentally from spectroscopic ellipsometry (*n* ≈ 2.0).

### Reporting summary

Further information on research design is available in the Nature Research Reporting Summary linked to this article.

## Supplementary information

Supplementary Information

Solar Cells Reporting Summary

## Data Availability

The data that support the findings of this study are available from the corresponding author upon reasonable request.
