# Supplementary material for: High-specific-power flexible transition metal dichalcogenide solar cells
Source: Nat Commun. 2021 Dec 9;12:7034. doi: 10.1038/s41467-021-27195-7 (PMC8660876; doi:10.1038/s41467-021-27195-7)
Supplement: Supplementary file 1 — Supplementary Information [file 41467_2021_27195_MOESM1_ESM.pdf]

Supplementary Information

High-specific-power flexible transition metal dichalcogenide solar cells

Koosha Nassiri Nazif,[1†] Alwin Daus,[1†] Jiho Hong,[2,3] Nayeun Lee,[2,3] Sam Vaziri,[1] Aravindh Kumar,[1] Frederick Nitta,[1] Michelle E. Chen,[3] Siavash Kananian,[1] Raisul Islam,[1] Kwan-Ho Kim,[4,5] Jin-Hong Park,[4,6] Ada S. Y. Poon,[1] Mark L. Brongersma,[2,3,7] Eric Pop,[1,3] and Krishna C. Saraswat[1,3*]

[1]Dept. of Electrical Engineering, Stanford University, Stanford, CA 94305, USA

[2]Geballe Laboratory for Advanced Materials, Stanford University, Stanford, CA 94305, USA

[3]Dept. of Materials Science and Engineering, Stanford University, Stanford, CA 94305, USA

[4]Dept. of Electrical and Computer Engineering, Sungkyunkwan University, Suwon 16419, Korea

[5]Dept. of Electrical and Systems Engineering, University of Pennsylvania, Philadelphia, PA 19104, USA

[6]SKKU Advanced Inst. of Nanotechnology (SAINT), Sungkyunkwan University, Suwon 16419, Korea

[7]Dept. of Applied Physics, Stanford University, Stanford, CA 94305, USA

[†]These authors contributed equally.

*corresponding author email: saraswat@stanford.edu

Supplementary Note 1. Detailed fabrication process including transfer procedure

We follow a recently developed fabrication and transfer approach,[1] where we perform the initial fabrication steps on a rigid Si/SiO_2 substrate followed by the release of patterned electrodes and the transition metal dichalcogenide (TMD) embedded into an ultrathin (~5 µm) flexible polyimide (PI) substrate (Supplementary Fig. 1). This technique is advantageous for vertical device architectures as it eliminates large steps in surface topography leading to a flat surface for the subsequent transfer of transparent graphene top electrodes.

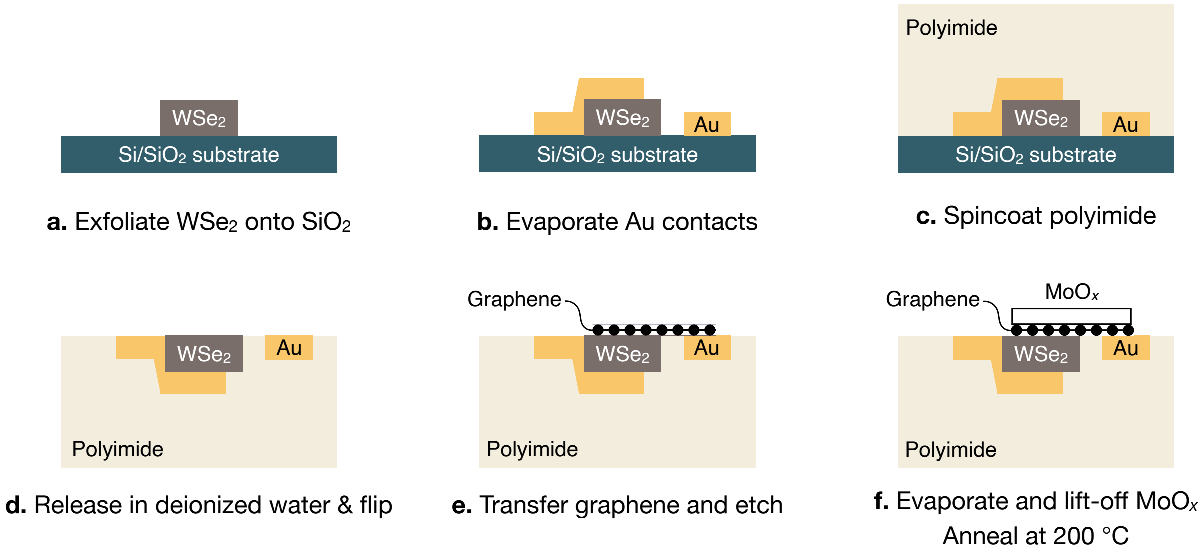

Supplementary Figure 1 | Flexible solar cell fabrication process. a, Exfoliated WSe_2 flake on Si/SiO_2 substrate. **b,** Deposition of Au bottom contact and contact pads. **c,** Polyimide spin-coating. **d,** Free-standing polyimide substrate (flipped) after release in deionized water as in Ref. 1. **e,** Graphene transfer and etching. **f,** MoO_x evaporation, patterning and anneal.

The detailed process steps are described below and the summarized process sequence is schematically shown in Supplementary Fig. 1. First, 90 nm of SiO_2 was grown on a bulk silicon wafer by dry thermal oxidation at 1100 °C. The wafers were then manually cut into ~2 cm × 2 cm pieces. Undoped (per manufacturer's description) WSe_2 flakes were mechanically exfoliated from the bulk crystal (2D Semiconductors) onto low-residue thermal release tape (Nitto Denko REVALPHA). They were subsequently transferred from the tape onto the Si/SiO_2 substrate using a WF film (Gel-Film®, WF-20-X4). Next, we spin-coated a lift-off layer LOL 2000 (3000 rpm, 60 s) on our substrates and baked it on a hot plate at 200 °C for 5 minutes. This was followed by spin-coating of Shipley 3612 photoresist (5500 rpm, 30 s) and baking on a hot plate at 90 °C for 1 minute. The lift-off layer/photoresist stack was then patterned by photolithography on a direct write lithography tool (Heidelberg MLA 150, dose:

90 mJ cm^{-2}, defocus: -2) and developed (MF-26A, 32 s). 135 nm of Au was deposited on top using electron-beam evaporation (AJA International). After a lift-off process similar as in Ref. 1, patterned Au bottom contacts and metal pads were left behind on top of the flakes and the substrate. The use of Au without any additional sticking layer enables us to later pick up the metal structures and TMD because no covalent bonds with the silicon dioxide substrate are formed (more details in Ref. 1).

The PI (PI-2610, HD MicroSystems) was spin-coated on top of the electrodes and TMD conformally covering all structures, baked at 90 °C and 150 °C for each 90 s, and finally cured at 250 °C in nitrogen ambient for 30 minutes. After that, all structures embedded into PI can be easily released from the silicon substrate by agitation and gentle mechanical force with a tweezer in deionized (DI) water without any discernable damage (more details in Ref. 1). After flipping the substrate, we temporarily attach it again to a silicon carrier with poly(methyl methacrylate) (PMMA) to perform the graphene transfer.

Graphene was grown via chemical vapor deposition at 1060 °C in an AIXTRON Black Magic furnace on commercial Cu foils with large preferentially 100 oriented grains (JX Mining, 99.9% purity HA-V2 treated rolled copper foil), which were surface cleaned in glacial acetic acid and commercial thin film nickel etchant (Transene, Nickel Etchant TFB) solution prior to growth.[2] After growth, the graphene was covered with PMMA via spin-coating (495-A2, 2500 rpm, 60 s, bake 140 °C for 45 s; 950-A4, 2000 rpm, 60 s, bake 80 °C 5 min). The backside of the Cu substrate was reactive ion etched in O_2 plasma (Oxford 80 RIE, 20 W, 10 mTorr, 40 sccm, 60 s). Then, the Cu/graphene/PMMA stack was dropped on top of $FeCl_3$ floating on the surface to etch the Cu from the backside. After that the graphene supported by PMMA was transferred by a glass slide into DI water (2 times) followed by diluted $HCl:H_2O_2$ solution (20:1:1 DI water:37% HCl:30% H_2O_2) and a final DI water beaker staying in each bath for 10 min. Then the graphene/PMMA layer was picked up by the supported PI substrate and the whole stack was allowed to gradually heat to 130 °C on a hotplate for drying. Finally, all PMMA (on top of graphene and below PI) was dissolved in toluene (2 hours) followed by acetone and iso-propanol soaks for each 5 min. To perform optical lithography on the free-standing PI substrates, we attached them to silicon carrier pieces prior to spin-coating with a few drops of photoresist (MEGAPOSIT™ SPR220-3) followed by nitrogen blow drying to flatten the substrate and remove excess photoresist and solvent, which was then baked on a hotplate at 90 °C for 5 minutes. The same photoresist was used for the optical lithography (spin-coating 3000 rpm, 30 s, bake 115 °C 90 s, exposure dose 250 mJ cm^{-2} and defocus -2, post-exposure bake 115 °C for 90 s, develop in MF-26A 75 s). We then etched graphene to remove it in undesired areas by reactive ion etching with the same parameters as mentioned above.

For the purpose of doping, passivation and anti-reflection coating, 10 nm of patterned MoO_x was deposited on top by e-beam evaporation (Kurt J. Lesker) from a MoO_3 pellet (Advanced Chemicals, 99.95% purity)

at a rate of 0.3 Å s^{-1}, using the same photolithography and lift-off processes as described for the graphene etch and Au contact definition, respectively. Afterwards, we annealed the samples on a hot plate at 200 °C in ambient air for 10 minutes in order to further oxidize MoO_x and therefore increase its work function, resulting in improved surface charge-transfer doping.[3]

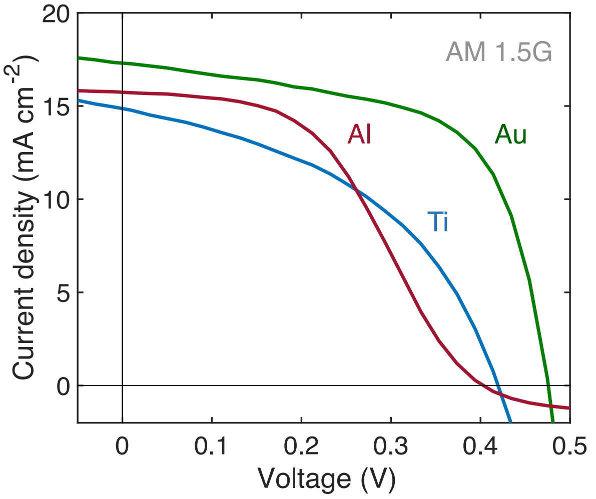

Supplementary Figure 2 | Performance of flexible WSe_2 solar cells having different back contact metals. Typical *J–V* characteristics of flexible WSe_2 solar cells having Al, Ti or Au back contact, under AM 1.5G illumination. Ti and Al, despite their lower work functions compared to Au, lead to worse performance, most probably due to their reactive nature and therefore forming poor interfaces with WSe_2. Ti and Al are known to be highly reactive/oxidizing, especially when in contact with TMDs, leading to the formation of AlO_x (~5 nm)[4] at the Al–WSe_2 interface and TiO_x + Ti_xSe_y at the Ti–WSe_2 interface.[5] These defective interfaces result in high levels of charge carrier recombination and therefore decreased open-circuit voltage and short-circuit current. Insulating AlO_x also induces high series resistance as well as a strong roll-over effect in Al-contact solar cells. Note that an AlO_x interlayer with reduced thickness (1-2 nm) could avoid these adverse effects while providing n-type doping as discussed in the manuscript.

Supplementary Note 2. Doping and passivation effects of MoO_x

The doping/passivation effect of MoO_x on graphene and WSe_2 was analyzed by Raman spectroscopy (Supplementary Table 1 and Supplementary Fig. 3a-b). We find small peak shifts towards higher wavenumber for graphene after MoO_x deposition, which has been previously reported to be a result of p-type doping and an increase of graphene's work function.[6] The WSe_2 peaks show no clearly discernable changes in peak position given the ~0.3 cm^{-1} detection limit of our Raman instrument, which is similar to the average shifts and standard deviations extracted here. This means that the bulk of WSe_2 (probed by Raman) is not affected by MoO_x. However, a small interface doping effect due to passivation of trap states at the graphene–WSe_2 interface could be associated with the slight red-shift of the Raman peaks.[7,8]

Supplementary Table 1 | Raman peak shifts upon deposition of MoO_x. A total of 12 spots on several WSe_2 flakes were measured. Average Raman peak positions (cm^{-1}) for WSe_2 and graphene before and after coating with MoO_x are compared in this table showing small peak shifts. The error bar represents the standard deviation.

**Peak**	**Before MoO_x**	**After MoO_x**
**Graphene: G**	1594.0 ± 3.9	1597.7 ± 5.9
**Graphene: 2D**	2689.2 ± 5.2	2702.0 ± 3.5
**WSe_2: E/A**	247.0 ± 0.2	246.7 ± 0.5
**WSe_2: 2LA(M)**	257.4 ± 0.3	257.2 ± 0.3

Based on the location of G and 2D peaks in our Raman measurements as well as previous literature reports on graphene doping,[6,9] we estimate carrier densities of ~6.5×10^{12} cm^{-2} and ~1.4×10^{13} cm^{-2} for air-exposed and MoO_x-capped graphene, respectively. The graphene Fermi level (referenced to the Dirac point) and carrier density are related according to the following equation:[10]

$$E_F(n) = \hbar v_F \sqrt{\pi n}$$

where n is the carrier density, E_F is the graphene Fermi level, $\hbar$ is the reduced Planck's constant and v_F is the graphene Fermi velocity (1.15×10^8 cm s^{-1}).[10] Using the above-mentioned carrier density values before and after MoO_x capping, we calculate a Fermi level downshift of 0.16 eV. This increase in Gr work function and therefore Gr–WSe_2 built-in potential along with the MoO_x surface passivation effects lead to a V_{OC} improvement of ~50 mV (Supplementary Fig. 3c).

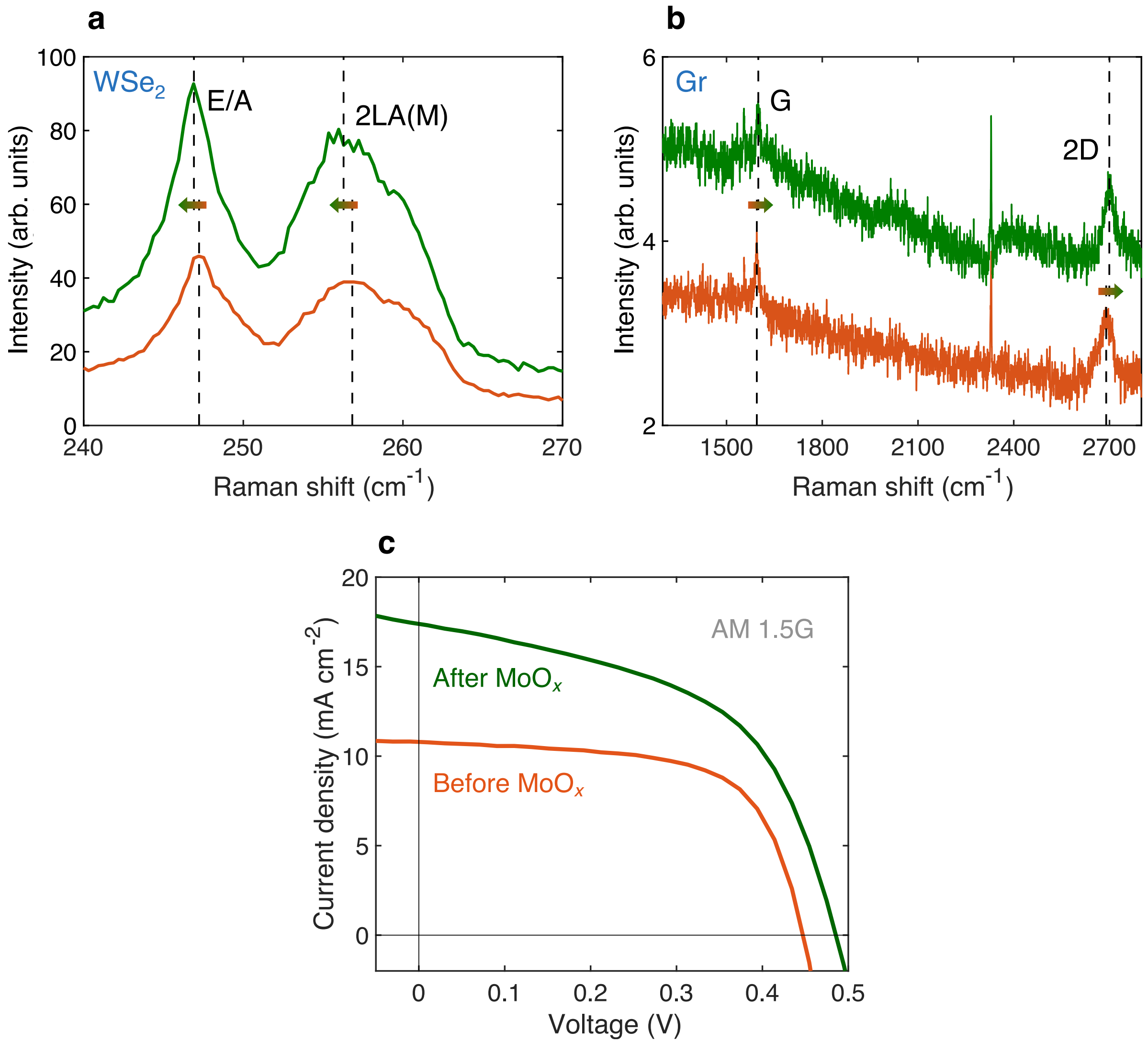

Supplementary Figure 3 | Performance-improvements by MoO_x. Raman spectroscopy of **a,** WSe_2 and **b,** Gr in the Gr–WSe_2–Au–PI stack before and after MoO_x deposition. Note, the narrow peak at ~2330 cm^{-1} can be associated with N_2 vibrations from ambient air.[11,12] **c,** *J*–*V* characteristics of an example Au–WSe_2–Gr vertical solar cell before and after 10 nm MoO_x capping. PCE is significantly improved after MoO_x capping due to doping, passivation and anti-reflection effects of MoO_x.

We also observe a significant J_{SC} boost of ~60% after 10 nm MoO_x capping (Supplementary Fig. 3c). Our optical simulation (Supplementary Fig. 9d) shows that MoO_x anti-reflection coating is responsible for ~7% of this boost (4.4% J_{SC} improvement). The remaining J_{SC} enhancement can be explained by doping and passivation effects of MoO_x and how they improve the drift length and carrier collection probability.

As discussed in the manuscript, the depletion region covers the entire depth of WSe_2. Usually, unity carrier collection probability is assumed for the depletion region, i.e. all photogenerated carriers in the depletion region are collected by the contacts. However, this is not necessarily true for materials like WSe_2 that have

a relatively low cross-plane mobility and carrier lifetime. To calculate the collection probability in the depletion region, the concept of drift length (similar to diffusion length) is used, which is directly proportional to the built-in potential and carrier lifetime:[13]

$$L_{drift} = \mu\tau V_{bi}/W$$

where μ is the carrier mobility, τ is the carrier lifetime, V_{bi} is the built-in potential, and W is the depletion width (WSe_2 thickness in this case). MoO_x capping increases both V_{bi} and τ, and therefore L_{drift}; doping graphene p-type and therefore increasing its work function enhances V_{bi}, and passivating the top surface of the solar cell improves τ.

Average collection probability (CP) of photogenerated carriers in the depletion region can be estimated by the following equation, where α is the absorption coefficient:[14]

$$\mathrm{CP} \approx \frac{\alpha L_{drift}}{1+ \alpha L_{drift}}$$

By increasing L_{drift} via doping and passivation, MoO_x increases the average collection probability and therefore J_{SC}, as detailed below.

Before MoO_x capping: Assuming a cross-plane WSe_2 mobility of 0.01 $cm^2\ V^{-1}\ s^{-1}$,[15] a carrier lifetime of 20 ns for unpassivated WSe_2,[16] an AM 1.5G-weighted-average absorption coefficient of 0.023 nm^{-1},[17] and a built-in potential of ~0.45 eV, we estimate drift length and collection probability of **45 nm** and **51%**, respectively, which match well with the extracted average internal quantum efficiency (IQE) of **56%**, calculated by dividing the measured J_{SC} (Supplementary Fig. 3c) by maximum attainable J_{SC} derived from optical simulations (Supplementary Fig. 9d).

After MoO_x capping: The built-in potential is increased to ~0.61 eV. Carrier lifetimes of up to 611 ns have been reported for multilayer TMDs.[18] Back-calculating from IQE of **87%** (extracted in the same way as above), a drift length of **300 nm** and a lifetime of **98 ns** are estimated.

Therefore the ~56% J_{SC} boost after MoO_x capping (excluding the anti-reflection effect) can be explained by the ~56% improvement in carrier collection probability, caused by the built-in potential increase from ~0.45 V to ~0.61 V (doping effect) and carrier lifetime enhancement from ~20 ns and ~98 ns (passivation effect).

Supplementary Note 3. Specific power calculation

The specific power (P_S) was calculated based on the solar cell efficiency and the areal weight densities of all materials in the solar cell stack including the PI substrate. Incident power is P_{in} = 100 mW cm^{-2}, which corresponds to one-sun illumination. We can then calculate the maximum output power P_{max} (unit: mW cm^{-2}) as follows:

$$P_{max} = J_{SC} \cdot V_{OC} \cdot FF = PCE \cdot P_{in}$$

where J_{SC}, V_{OC}, FF and PCE are the short-circuit current density, open-circuit voltage, fill factor and power conversion efficiency, respectively. Then, we sum up the areal mass densities of all materials in the solar cell stack including the substrate by using the volumetric mass density multiplied by the respective material thickness.

Supplementary Table 2 | Areal mass density of our solar cells. The volumetric densities of all materials were taken from literature. For MoO_x the volumetric mass density of MoO_3 was assumed.

**Peak**	**PI**	**Au**	**WSe_2**	**Gr**	**MoO_x**	**Total**
**Thickness (nm)**	5000	135	200	0.3	10	
**Volumetric mass density (g m^{-3})**	1.4×10^6 (19)	1.93×10^7 (20)	9.28×10^6 (21)	2.27×10^6 (22)	4.69×10^6 (23)	
**Areal mass density (g m^{-2})**	7	2.61	1.86	0.00068	0.047	11.52

We calculate P_S (unit: W g^{-1}) by dividing P_{max} by the total area mass density from Supplementary Table 2. With P_{max} = 5.1 mW cm^{-2} (see Fig. 1f) we obtain P_S = 4.4 W g^{-1}. A simple approach to reduce P_S would be thinning down the substrate to ~1 µm, which is the approximate substrate thickness in some of the works with highest P_S achieved so far,[24–26] leading to a value of ~8.6 W g^{-1}. A PCE of ~27% can be practically achieved[27] in an optimized TMD single-junction (Supplementary Note 5), further increasing the P_S to ~46 W g^{-1}. We included these projections in Fig. 5 to emphasize the great potential of TMDs for high-specific-power photovoltaics.

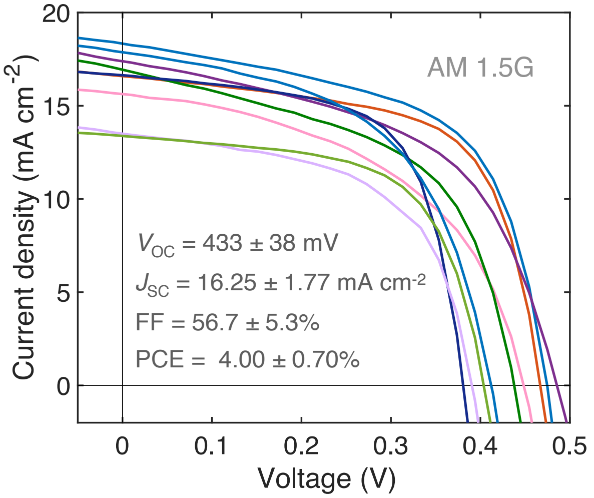

Supplementary Figure 4 | Reproducibility. *J–V* measurements of 9 different flexible WSe_2 solar cells under AM 1.5G illumination, showing similar characteristics. The numbers represent mean ± standard deviation for each parameter.

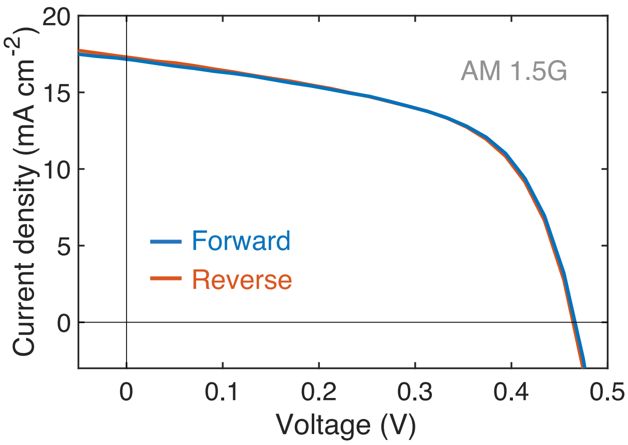

Supplementary Figure 5 | Forward and backward scans. J-V characteristics under one-sun illumination show no hysteresis in forward/reverse voltage sweeps.

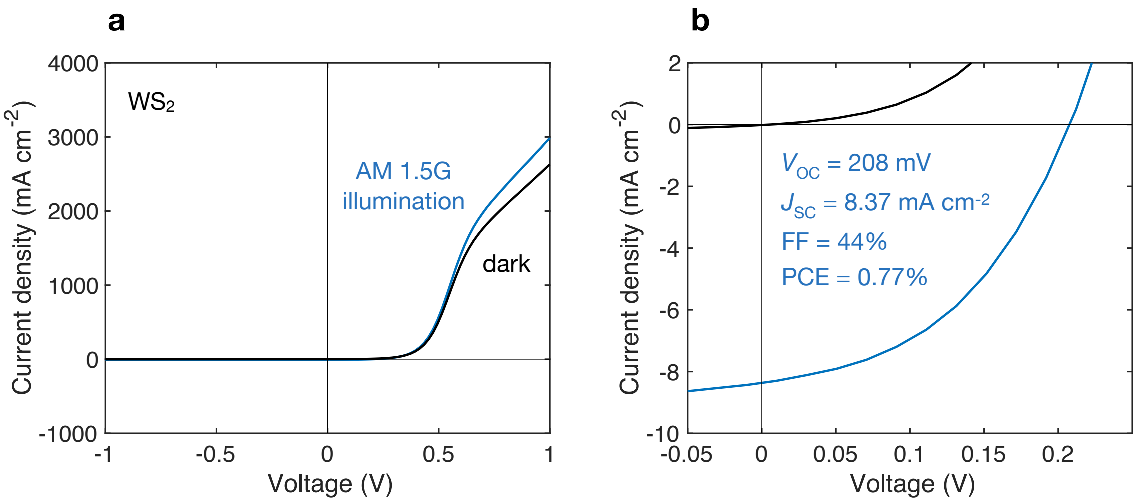

Supplementary Figure 6 | Flexible WS_2 solar cells. **a-b,** *J*–*V* characteristics of flexible WS_2 solar cells having the exact same design as WSe_2 cells discussed in the main text (Fig. 1b), in dark and under one-sun illumination. **b,** a zoom-in view of the photovoltaic region. WS_2 solar cells show the same *J*–*V* characteristics as WSe_2 cells, however with lower performance. Similar to WSe_2, Au pins near the midgap of WS_2.[28] Given the higher electron affinity and bandgap in WS_2,[29] this leads to a smaller built-in potential and therefore reduced V_{OC}, J_{SC}, and FF in fully depleted Gr–WS_2–Au solar cells. Further p-doping Gr (for example by using flame-deposited MoO_3)[6] is one way to achieve a similar built-in potential and therefore performance in WS_2 solar cells.

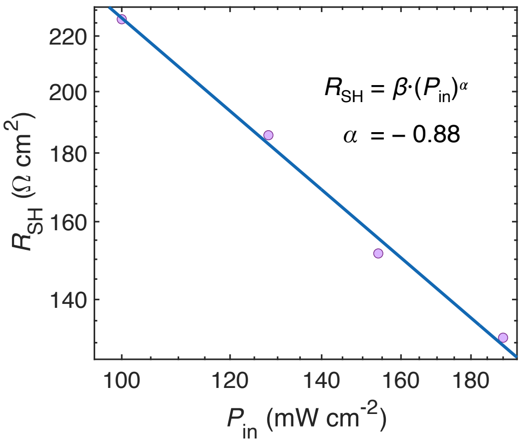

Supplementary Figure 7 | Photoshunting. Shunt resistance (R_{SH}) of a typical flexible WSe_2 solar cell at various incident power (P_{in}) intensities of AM 1.5G illumination. Shunt resistance decreases almost linearly with increasing incident power intensity due to increased minority carrier conductivity across the device under illumination, a phenomenon known as photoshunting.[30,31] Utilizing contacts with greater carrier selectivity or introducing a high built-in potential p-n homojunction could reduce or eliminate the photoshunting observed here. Symbols, measurements; line, power law fit.

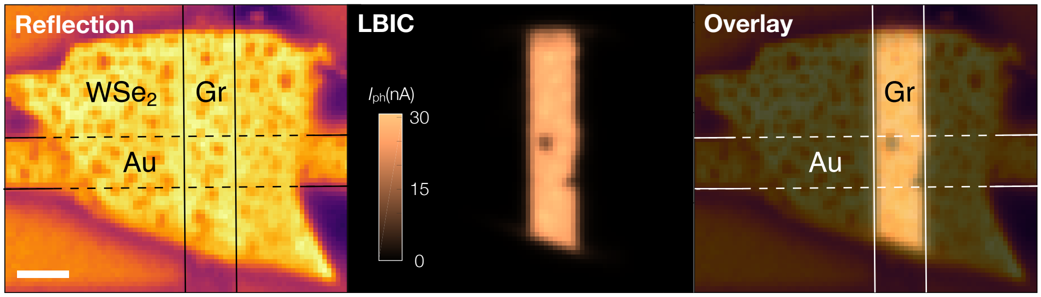

Supplementary Figure 8 | Photocurrent mapping. Spatial maps of reflection, photocurrent, and reflection/photocurrent overlay for a flexible Au–WSe_2–Gr solar cell measured at λ = 530 nm. Current generation occurs only in the WSe_2 region under the Gr contact, which can be used to accurately define the active area of the solar cell. No photocurrent generation is observed at the Au–WSe_2 back diode. Scale bar, 10 μm.

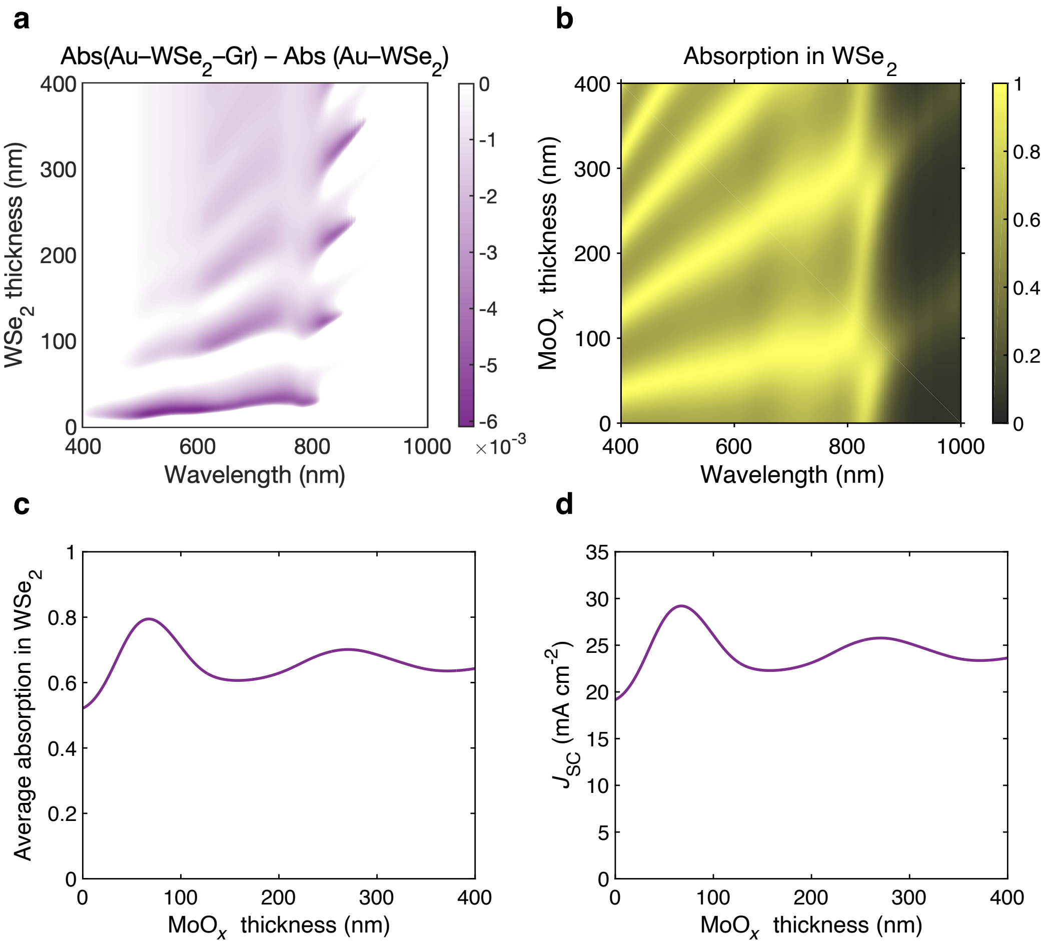

Supplementary Figure 9 | Effects of Gr and MoO$_x$ on optical absorption. a, Absorption reduction by graphene. The difference in total absorption between the Au–WSe$_2$ and Au–WSe$_2$–Gr stacks, showing that adding graphene on top slightly reduces the total absorption in the ~500–800 nm wavelength spectrum for WSe$_2$ thicknesses around 200 nm, in agreement with experimental measurements (Fig. 3c). **b–d,** Anti-reflection coating effects of MoO$_x$. **b,** Absorption spectrum and **c,** average absorption of WSe$_2$ in the Au–WSe$_2$–Gr–MoO$_x$ stack as a function of MoO$_x$ thickness. WSe$_2$ thickness is 209 nm, similar to the device in Fig. 3c. **d,** Maximum short-circuit current density (J_{SC}) attainable from WSe$_2$ in the same stack, as a function of MoO$_x$ thickness, calculated by integrating Absorption(λ) × (spectral photon flux of AM1.5G spectrum at one-sun solar intensity) over the wavelength range of λ = 400–1000 nm, assuming unity internal quantum efficiency (IQE). This maximum J_{SC} value is slightly underestimated as absorption at wavelengths below 400 nm and above 1000 nm are not included due to lack of material data. An optimal MoO$_x$ thickness of ~70 nm can increase the average absorption in WSe$_2$ and similarly J_{SC} by ~50%, leading to a remarkable ~80% average absorption and ~30 mA cm^{-2} J_{SC} in WSe$_2$ solar cells.

Supplementary Note 4. Benchmarking

We performed an extensive literature review on flexible and light-weight solar cells with various absorber materials and calculated P_S based on information provided. Some works directly stated P_S or at least PCE and the areal mass density, while others did not explicitly provide information on the weight of their solar cells. In some cases, we calculated the weight based on the substrate and the solar cell layer stack if the substrate weight was small (sufficiently thin/low density substrate material) and therefore the weight of other materials was non-negligible. In other cases, we only used the substrate weight for estimating P_S if it appeared to dominate (thicker high-density substrate materials) and neglected the weight of other materials. Supplementary Table 3 lists all the works shown in Fig. 5 indicating how P_S was obtained.

Supplementary Table 3 | Literature reports on flexible and light-weight solar cells with notable specific power (P_S), along with their power conversion efficiencies (PCE). Unmarked P_S is directly taken from the literature reports. *P_S calculated based on the solar cell layer stack including the substrate. **P_S calculated only based on substrate weight or absorber material if free-standing. ***PCE corresponds to performance under AM 0 illumination (space).

**Reference**	**Technology**	**PCE (%)**	**P_S (W g^{-1})**
Kaltenbrunner et al.[32]	Perovskite	12	23
Kaltenbrunner et al.[33]	Organic	4	10
Söderström et al.[34]	Amorphous Si (a-Si)	8.8	3.3
Chirilă et al.[35]	CuInGaSe (CIGS)	18.7	3.3
Shiu et al.[36]	InP (III-V)	10.2	2.0
Romeo et al.[37]	CdTe (II-VI)	11.4	2.1
Fatemi et al.[38]	3-mil Si (c-Si)	17***	0.85
Fatemi et al.[38]	InGaP/GaAs/Ge (III-V)	26***	0.4
Zhao et al.[39]	Single-crystal Si (c-Si)	24.4	0.4
Zhao et al.[39]	Polycrystalline Si (poly-Si)	19.8	0.33
Bremaud et al.[40]	CuInGaSe (CIGS)	15	0.09
Kang et al.[24]	Perovskite	12.85	29.4
Zhang et al.[25]	PbS quantum dot	9.9	15.2
Liu et al.[41]	Perovskite	11.5	5
Lin et al.[42]	Amorphous Si (a-Si)	7.06	0.5**
Park et al.[26]	Organic	10.5	11.46

Reference	Technology	PCE (%)	P_S (W g^{-1})
Tavakoli et al.[43]	PbS quantum dot	7.1	12.3
Li et al.[44]	Perovskite	13	3.7**
Jinno et al.[45]	Organic	7.9	14*
Jia et al.[46]	Perovskite	18	1.3**
Lee et al.[47]	Perovskite	17.03	18.5*
Cardwell et al.[48]	GaInP/GaAs/GaInAs (III-V)	29.3***	3.8
Xie et al.[49]	Perovskite	13.32	4.16
Sun et al.[50]	Amorphous Si (a-Si)	5.6	1.382
Başol et al.[51]	$CuInSe_2$ (CIS)	9.3	1.133
Rance et al.[52]	CdTe (II-VI)	14.05	0.6**
Mahabaduge et al.[53]	CdTe (II-VI)	16.4	0.7**
Law et al.[54]	GaInP/GaInAs/Ge (III-V)	21***	2.067
Kim et al.[55]	GaAs (III-V)	15.2	5.9*
Gerthoffer et al.[56]	$Cu(In,Ga)Se_2$ (CIGS)	11.2	0.4**
Jeong et al.[57]	Single-crystal Si (c-Si)	13.7	5.88**
Das et al.[58]	Single-crystal Si (c-Si)	9	1.89
Hwang et al.[59]	Single-crystal Si (c-Si)	18.4	1.3*
Jean et al.[60]	Organic	2.2	6.11
Salavei et al.[61]	CdTe (II-VI)	10	1.98
Shahrjerdi et al.[62]	InGaP/(In)GaAs (III-V)	28.1	1.995
Cho et al.[63]	CdS/CdTe (II-VI)	13.56	0.254
Zhao et al.[64]	Organic	8.7	0.4
Li et al.[65]	Perovskite	14	1.96
Romeo et al.[66]	CdTe/CdS (II-VI)	11	2.6*
Liu et al.[67]	Organic	6.62	1.71
Chirilă et al.[68]	$Cu(In,Ga)Se_2$ (CIGS)	20.4	3.4*
Xu et al.[69]	Amorphous Si (a-Si)	9.7***	1.2
Mavlonov et al.[70]	$Cu(In,Ga)Se_2$ (CIGS)	9.1	0.7*
Qu et al.[71]	Organic	16.1	11.5**
Koo et al.[72]	Organic	15.2	10.8**
Garud et al.[73]	Polycrystalline Si (poly-Si)	15	0.06**
El-Atab et al.[74]	Single-crystal Si (c-Si)	19.1	0.24

Reference	Technology	PCE (%)	P_S (W g^{-1})
Augusto et al.[75]	Single-crystal Si (c-Si)	18.4	1.3**
Lee et al.[76]	Single-crystal Si (c-Si)	12.4	1*
El-Atab et al.[77]	Single-crystal Si (c-Si)	19	0.29
Xue et al.[78]	Single-crystal Si (c-Si)	12.3	9.33*
Tanabe et al.[79]	InAs/GaAs quantum dot (III-V)	10.5	0.6**
Wu et al.[80]	Dye-Sensitized (DSSC)	6.69	0.3**
Akama et al.[81]	WS_2 (TMD)	<0.7%	<0.04**

Supplementary Note 5. Realistic efficiency limits of WSe_2 solar cells

We performed realistic detailed balance calculations to determine the efficiency limits of ultrathin WSe_2 solar cells which can be achieved in practice with an optimized optical and electronic design. We started by the ideal Shockley–Queisser model[82] where all photons with energies above the band gap energy are absorbed and the only recombination mechanism is radiative, leading to an idealistic 33.4% efficiency limit.

Next, we adopted the more advanced Tiedje–Yablonovitch model,[83] where the measured spectral absorption coefficient of the absorber layer (WSe_2)[17] is used to calculate both absorption and radiative recombination. The model also includes free carrier absorption and Auger recombination (data from Ref. 84) in addition to radiative losses. This leads to a lower, more realistic efficiency limit which is dependent on the thickness of the absorber layer.

Lastly, we added the defect-assisted Shockley-Read-Hall (SRH) recombination to the Tiedje–Yablonovitch model to account for the varying material quality of TMDs, which is correlated with SRH recombination lifetime (τ_{SRH}); the higher the SRH lifetime, the higher the material quality. SRH lifetimes of up to 611 ns have been reported for multilayer TMDs, which, according to our calculations, corresponds to a realistic efficiency limit of ~27% occurring at a WSe_2 thickness of ~110 nm (Supplementary Fig. 10).

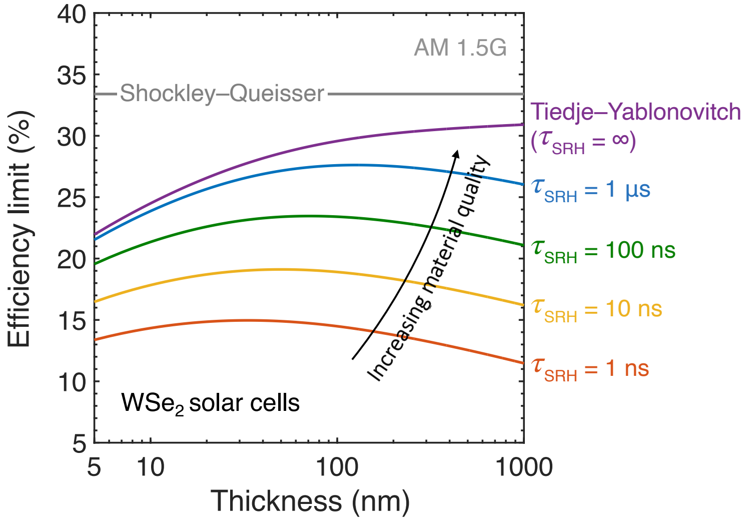

Supplementary Figure 10 | Efficiency limits of WSe_2 solar cells. Shockley–Queisser, Tiedje–Yablonovitch and modified Tiedje–Yablonovitch (including SRH recombination) efficiency limits of WSe_2 solar cells under AM 1.5G illumination. The realistic modified Tiedje–Yablonovitch model reveals maximum efficiencies practically attainable in ultrathin WSe_2 solar cells at different material quality levels (SRH lifetimes, τ_{SRH}) and their corresponding optimal thicknesses. SRH lifetimes of up to 611 ns have been reported for multilayer TMDs,[18] leading to a maximum efficiency of ~27% at an optimal thickness of ~110 nm.

Supplementary References:

1. Daus, A. *et al.* High-performance flexible nanoscale transistors based on transition metal dichalcogenides. *Nat. Electron.* **4,** 495–501 (2021).
2. Chen, M. E. *et al.* Graphene-based electromechanical thermal switches. *2D Mater*. **8,** 035055 (2021).
3. Ishikawa, R. *et al.* Effect of annealing on doping of graphene with molybdenum oxide. *Appl. Phys. Express* **11**, 45101 (2018).
4. Lu, C.-C., Lin, Y.-C., Yeh, C.-H., Huang, J.-C. & Chiu, P.-W. High mobility flexible graphene field-effect transistors with self-healing gate dielectrics. *ACS Nano* **6**, 4469–4474 (2012).
5. Schauble, K. *et al.* Uncovering the effects of metal contacts on monolayer MoS_2. *ACS Nano* **14**, 14798–14808 (2020).
6. Vaziri, S. *et al.* Ultrahigh doping of graphene using flame-deposited MoO_3. *IEEE Electron Device Lett.* **41**, 1592–1595 (2020).
7. Nassiri Nazif, K. *et al.* High-performance p–n junction transition metal dichalcogenide photovoltaic cells enabled by MoO_x doping and passivation. *Nano Lett.* **21**, 3443–3450 (2021).
8. Cai, L. *et al.* Rapid flame synthesis of atomically thin MoO_3 down to monolayer thickness for effective hole doping of WSe_2. *Nano Lett.* **17**, 3854–3861 (2017).
9. Das, A. *et al.* Monitoring dopants by Raman scattering in an electrochemically top-gated graphene transistor. *Nat. Nanotechnol.* **3**, 210–215 (2008).
10. Kim, S. *et al.* Direct measurement of the Fermi energy in graphene using a double-layer heterostructure. *Phys. Rev. Lett.* **108**, 116404 (2012).
11. Huang, N. *et al.* Full range characterization of the Raman spectra of organs in a murine model. *Opt. Express* **19**, 22892–22909 (2011).
12. Spizzirri, P. G., Fang, J.-H., Rubanov, S., Gauja, E. & Prawer, S. Nano-Raman spectroscopy of silicon surfaces. (2010).
13. Johnston, K. W. *et al.* Efficient Schottky-quantum-dot photovoltaics: the roles of depletion, drift, and diffusion. *Appl. Phys. Lett.* **92**, 122111 (2008).
14. Li, S. S. *Semiconductor Physical Electronics* (Springer, New York, 2006)
15. Massicotte, M. *et al.* Picosecond photoresponse in van der Waals heterostructures. *Nat. Nanotechnol.* **11**, 42–46 (2016).
16. Jakubowicz, A., Mahalu, D., Wolf, M., Wold, A. & Tenne, R. WSe_2: optical and electrical properties as related to surface passivation of recombination centers. *Phys. Rev. B* **40**, 2992–3000 (1989).
17. Kravets, V. G. Ellipsometry and optical spectroscopy of low-dimensional family TMDs. *Semicond. Phys. Quantum Electron. Optoelectron.* **20**, 284–296 (2017).

18. Went, C. M. *et al.* A new metal transfer process for van der Waals contacts to vertical Schottky-junction transition metal dichalcogenide photovoltaics. *Sci. Adv.* **5**, eaax6061 (2019).
19. Density of polyimide. Available at: https://www.yumpu.com/fr/document/view/2032698/pi-2600-series-low-stress-applications-hd-microsystems.
20. Density of gold (Au). Available at: http://chemistry.elmhurst.edu/vchembook/125Adensitygold.html.
21. Agarwal, M. K. & Wani, P. A. Growth conditions and crystal structure parameters of layer compounds in the series $Mo_{1-x}W_xSe_2$. *Mater. Res. Bull.* **14**, 825–830 (1979).
22. Density of graphene (Gr). Available at: https://www.americanelements.com/graphene-1034343-98-0.
23. Density of molybdenum trioxide (MoO_3). Available at: https://pubchem.ncbi.nlm.nih.gov/compound/Molybdenum-trioxide.
24. Kang, S. *et al.* Ultrathin, lightweight and flexible perovskite solar cells with an excellent power-per-weight performance. *J. Mater. Chem. A* **7**, 1107–1114 (2019).
25. Zhang, X., Öberg, V. A., Du, J., Liu, J. & Johansson, E. M. J. Extremely lightweight and ultra-flexible infrared light-converting quantum dot solar cells with high power-per-weight output using a solution-processed bending durable silver nanowire-based electrode. *Energy Environ. Sci.* **11**, 354–364 (2018).
26. Park, S. *et al.* Self-powered ultra-flexible electronics via nano-grating-patterned organic photovoltaics. *Nature* **561**, 516–521 (2018).
27. Jariwala, D., Davoyan, A. R., Wong, J. & Atwater, H. A. Van der Waals materials for atomically-thin photovoltaics: promise and outlook. *ACS Photonics* **4**, 2962–2970 (2017).
28. Park, W. *et al.* Complementary unipolar WS_2 field-effect transistors using Fermi-level depinning layers. *Adv. Electron. Mater.* **2**, 1500278 (2016).
29. Guo, Y. & Robertson, J. Band engineering in transition metal dichalcogenides: stacked versus lateral heterostructures. *Appl. Phys. Lett.* **108**, 233104 (2016).
30. Waldauf, C., Scharber, M. C., Schilinsky, P., Hauch, J. A. & Brabec, C. J. Physics of organic bulk heterojunction devices for photovoltaic applications. *J. Appl. Phys.* **99**, 104503 (2006).
31. Würfel, U., Cuevas, A. & Würfel, P. Charge carrier separation in solar cells. *IEEE J. Photovoltaics* **5**, 461–469 (2015).
32. Kaltenbrunner, M. *et al.* Flexible high power-per-weight perovskite solar cells with chromium oxide–metal contacts for improved stability in air. *Nat. Mater.* **14**, 1032–1039 (2015).
33. Kaltenbrunner, M. *et al.* Ultrathin and lightweight organic solar cells with high flexibility. *Nat. Commun.* **3**, 770 (2012).

34. Söderström, T., Haug, F.-J., Terrazzoni-Daudrix, V. & Ballif, C. Optimization of amorphous silicon thin film solar cells for flexible photovoltaics. *J. Appl. Phys.* **103**, 114509 (2008).
35. Chirilă, A. *et al.* Highly efficient Cu(In,Ga)Se_2 solar cells grown on flexible polymer films. *Nat. Mater.* **10**, 857–861 (2011).
36. Shiu, K.-T., Zimmerman, J., Wang, H. & Forrest, S. R. Ultrathin film, high specific power InP solar cells on flexible plastic substrates. *Appl. Phys. Lett.* **95**, 223503 (2009).
37. Romeo, A. *et al.* High-efficiency flexible CdTe solar cells on polymer substrates. *Sol. Energy Mater. Sol. Cells* **90**, 3407–3415 (2006).
38. Fatemi, N. S., Pollard, H. E., Hou, H. Q. & Sharps, P. R. Solar array trades between very high-efficiency multi-junction and Si space solar cells. in *Conference Record of the Twenty-Eighth IEEE Photovoltaic Specialists Conference - 2000 (Cat. No.00CH37036)* 1083–1086 (2000).
39. Zhao, J., Wang, A., Green, M. A. & Ferrazza, F. 19.8% efficient "honeycomb" textured multicrystalline and 24.4% monocrystalline silicon solar cells. *Appl. Phys. Lett.* **73**, 1991–1993 (1998).
40. Bremaud, D., Rudmann, D., Bilger, G., Zogg, H. & Tiwari, A. N. Towards the development of flexible CIGS solar cells on polymer films with efficiency exceeding 15%. in *Conference Record of the Thirty-first IEEE Photovoltaic Specialists Conference, 2005.* 223–226 (2005).
41. Liu, Z., You, P., Xie, C., Tang, G. & Yan, F. Ultrathin and flexible perovskite solar cells with graphene transparent electrodes. *Nano Energy* **28**, 151–157 (2016).
42. Lin, Q. *et al.* High performance thin film solar cells on plastic substrates with nanostructure-enhanced flexibility. *Nano Energy* **22**, 539–547 (2016).
43. Tavakoli, M. M. *et al.* Efficient, flexible, and ultra-lightweight inverted PbS quantum dots solar cells on all-CVD-growth of parylene/graphene/oCVD PEDOT substrate with high power-per-weight. *Adv. Mater. Interfaces* **7**, 2000498 (2020).
44. Li, H. *et al.* Ultraflexible and biodegradable perovskite solar cells utilizing ultrathin cellophane paper substrates and TiO_2/Ag/TiO_2 transparent electrodes. *Sol. Energy* **188**, 158–163 (2019).
45. Jinno, H. *et al.* Stretchable and waterproof elastomer-coated organic photovoltaics for washable electronic textile applications. *Nat. Energy* **2**, 780–785 (2017).
46. Jia, C. *et al.* Highly flexible, robust, stable and high efficiency perovskite solar cells enabled by van der Waals epitaxy on mica substrate. *Nano Energy* **60**, 476–484 (2019).
47. Lee, G. *et al.* Ultra-flexible perovskite solar cells with crumpling durability: toward a wearable power source. *Energy Environ. Sci.* **12**, 3182–3191 (2019).
48. Cardwell, D. *et al.* Very high specific power ELO solar cells (>3 kW/kg) for UAV, space, and portable power applications. in *2017 IEEE 44th Photovoltaic Specialist Conference (PVSC)* 3511–

3513 (2017).
49. Xie, M. *et al.* Super-flexible perovskite solar cells with high power-per-weight on 17 μm thick PET substrate utilizing printed Ag nanowires bottom and top electrodes. *Flex. Print. Electron.* **4**, 34002 (2019).
50. Sun, X. *et al.* Firmly standing three-dimensional radial junctions on soft aluminum foils enable extremely low cost flexible thin film solar cells with very high power-to-weight performance. *Nano Energy* **53**, 83–90 (2018).
51. Başol, B. M., Kapur, V. K., Leidholm, C. R., Halani, A. & Gledhill, K. Flexible and light weight copper indium diselenide solar cells on polyimide substrates. *Sol. Energy Mater. Sol. Cells* **43**, 93–98 (1996).
52. Rance, W. L. *et al.* 14%-efficient flexible CdTe solar cells on ultra-thin glass substrates. *Appl. Phys. Lett.* **104**, 143903 (2014).
53. Mahabaduge, H. P. *et al.* High-efficiency, flexible CdTe solar cells on ultra-thin glass substrates. *Appl. Phys. Lett.* **106**, 133501 (2015).
54. Law, D. C. *et al.* Lightweight, flexible, high-efficiency III-V multijunction cells. in *2006 IEEE 4th World Conference on Photovoltaic Energy Conference* **2**, 1879–1882 (2006).
55. Kim, J. *et al.* Ultra-thin flexible GaAs photovoltaics in vertical forms printed on metal surfaces without interlayer adhesives. *Appl. Phys. Lett.* **108**, 253101 (2016).
56. Gerthoffer, A. *et al.* CIGS solar cells on flexible ultra-thin glass substrates: characterization and bending test. *Thin Solid Films* **592**, 99–104 (2015).
57. Jeong, S., McGehee, M. D. & Cui, Y. All-back-contact ultra-thin silicon nanocone solar cells with 13.7% power conversion efficiency. *Nat. Commun.* **4**, 2950 (2013).
58. Das, S. *et al.* A leaf-inspired photon management scheme using optically tuned bilayer nanoparticles for ultra-thin and highly efficient photovoltaic devices. *Nano Energy* **58**, 47–56 (2019).
59. Hwang, I. *et al.* Effective photon management of non-surface-textured flexible thin crystalline silicon solar cells. *Cell Reports Phys. Sci.* **1**, 100242 (2020).
60. Jean, J., Wang, A. & Bulović, V. In situ vapor-deposited parylene substrates for ultra-thin, lightweight organic solar cells. *Org. Electron.* **31**, 120–126 (2016).
61. Salavei, A. *et al.* Flexible CdTe solar cells on polyimide and flexible glass substrates. *Proc. EU PVSEC 2015* 1356–1357 (2015).
62. Shahrjerdi, D. *et al.* Ultralight high-efficiency flexible InGaP/(In)GaAs tandem solar cells on plastic. *Adv. Energy Mater.* **3**, 566–571 (2013).
63. Cho, E., Kang, Y., Kim, D. & Kim, J. Post-growth process for flexible CdS/CdTe thin film solar cells with high specific power. *Opt. Express* **24**, A791–A796 (2016).

64. Zhao, B. *et al.* Flexible polymer solar cells with power conversion efficiency of 8.7%. *J. Mater. Chem. C* **2**, 5077–5082 (2014).

65. Li, Y. *et al.* High-efficiency robust perovskite solar cells on ultrathin flexible substrates. *Nat. Commun.* **7**, 10214 (2016).

66. Romeo, A., Arnold, M., Bätzner, D. L., Zogg, H. & Tiwari, A. N. Development of high efficiency flexible solar cells. in *PV in Europe - From PV Technology to Energy Solutions* **1**, 377–381 (2002).

67. Liu, Y. *et al.* Highly flexible and lightweight organic solar cells on biocompatible silk fibroin. *ACS Appl. Mater. Interfaces* **6**, 20670–20675 (2014).

68. Chirilă, A. *et al.* Potassium-induced surface modification of Cu(In,Ga)Se_2 thin films for high-efficiency solar cells. *Nat. Mater.* **12**, 1107–1111 (2013).

69. Xu, X. *et al.* High efficiency ultra lightweight a-Si:H/a-SiGe:H/a-SiGe:H triple-junction solar cells on polymer substrate using roll-to-roll technology. in *2008 33rd IEEE Photovoltaic Specialists Conference* 1–6 (2008).

70. Mavlonov, A. *et al.* Superstrate-type flexible and bifacial Cu(In,Ga)Se_2 thin-film solar cells with In_2O_3:SnO_2 back contact. *Sol. Energy* **211**, 725–731 (2020).

71. Qu, T.-Y. *et al.* Biomimetic electrodes for flexible organic solar cells with efficiencies over 16%. *Adv. Opt. Mater.* **8**, 2000669 (2020).

72. Koo, D. *et al.* Flexible organic solar cells over 15% efficiency with polyimide-integrated graphene Electrodes. *Joule* **4**, 1021–1034 (2020).

73. Garud, S. *et al.* Toward high solar cell efficiency with low material usage: 15% efficiency with 14 μm polycrystalline silicon on glass. *Sol. RRL* **4**, 2000058 (2020).

74. El-Atab, N., Shamsuddin, R., Bahabry, R. & Hussain, M. M. High-Efficiency corrugated monocrystalline silicon solar cells with multi-directional flexing capabilities. in *2019 IEEE 46th Photovoltaic Specialists Conference (PVSC)* 1499–1501 (2019).

75. Augusto, A., Tyler, K., Herasimenka, S. Y. & Bowden, S. G. Flexible modules using <70 μm thick silicon solar cells. *Energy Procedia* **92**, 493–499 (2016).

76. Lee, S.-M. *et al.* Printable nanostructured silicon solar cells for high-performance, large-area flexible photovoltaics. *ACS Nano* **8**, 10507–10516 (2014).

77. El-Atab, N., Khan, S. M. & Hussain, M. M. Flexible high-efficiency corrugated monocrystalline silicon solar cells for application in small unmanned aerial vehicles for payload transportation. *Energy Technol.* **8**, 2000670 (2020).

78. Xue, M. *et al.* Free-standing 2.7 μm thick ultrathin crystalline silicon solar cell with efficiency above 12.0%. *Nano Energy* **70**, (2020).

79. Tanabe, K., Watanabe, K. & Arakawa, Y. Flexible thin-film InAs/GaAs quantum dot solar cells.

Appl. Phys. Lett. **100**, 192102 (2012).

80. Wu, J. *et al.* A Large-area light-weight dye-sensitized solar cell based on all titanium substrates with an efficiency of 6.69% outdoors. *Adv. Mater.* **24**, 1884–1888 (2012).
81. Akama, T. *et al.* Schottky solar cell using few-layered transition metal dichalcogenides toward large-scale fabrication of semitransparent and flexible power generator. *Sci. Rep.* **7**, 11967 (2017).
82. Shockley, W. & Queisser, H. J. Detailed balance limit of efficiency of p-n junction solar cells. *J. Appl. Phys.* **32**, 510–519 (1961).
83. Tiedje, T., Yablonovitch, E., Cody, G. D. & Brooks, B. G. Limiting efficiency of silicon solar cells. *IEEE Trans. Electron Devices* **31**, 711–716 (1984).
84. Piprek, J. Efficiency droop in nitride-based light-emitting diodes. *Phys. status solidi* **207**, 2217–2225 (2010).
